# Editorial: Mechanical behavior of hydrogels for soft tissue replacement and regeneration

**DOI:** 10.3389/fbioe.2023.1254076

**Published:** 2023-07-25

**Authors:** Silvia Todros, Miguel Castilho, Daniel M. Espino, Piero G. Pavan

**Affiliations:** ^1^ Department of Industrial Engineering, University of Padova, Padova, Italy; ^2^ Department of Biomedical Engineering, Eindhoven University of Technology, Eindhoven, Netherlands; ^3^ Department of Mechanical Engineering, University of Birmingham, Birmingham, United Kingdom

**Keywords:** hydrogel, mechanical properties, soft tissue, bio fabrication, tissue engineering

Soft tissue repair and regeneration is a field of bioengineering focused on the development of bioactive substitutes capable of restoring the physiological function of damaged and diseased tissues. Biomaterials play a crucial role in this field, serving as either bio-inert materials that integrate well into living tissues with minimal biological response, or as bioactive materials that promote new tissue growth while slowly degrading in the body. In most tissue repair and regeneration applications, several mechanical aspects come into play, such as *in vivo* loading conditions, time-dependent mechanical effects and mechanotransduction. Accordingly, the characteristics of the chosen biomaterials have a significant impact on the final mechanical performance at different size scales, ranging from cellular up to tissue/organ level.

Hydrogels exhibit great potential as biomaterials for soft tissue replacement and regeneration due to their unique physicochemical and mechanical properties. They possess the ability to absorb large amounts of water, show low elastic modulus and viscoelastic behavior. Because of their structural similarity with the extracellular matrix (ECM), engineered hydrogels can resemble native ECM, providing an ideal environment for cell survival. Moreover, they can also be used as a bioink, which is seeded with living cells for the 3D bioprinting of artificial tissues and organs, or as injectable substances able to adapt to the shape of tissue defects.

Injectable hydrogels find frequent use in cartilage defect repair (Zhu et al.), as they can conform to irregular cartilage surfaces, and offer excellent biocompatibility and tunable physicochemical properties. Both natural and synthetic polymers can be adopted for this purpose, and different cross-linking methods allow for the customization of hydrogel structure, resulting in high water content, good cell interaction and improved mechanical properties. The ability to withstand high compressive loads and to reduce friction are fundamental requirements for cartilage tissue substitutes. However, hydrogels currently exhibit compressive stiffness one to two orders of magnitude lower than that of articular cartilage. Additionally, the rapid degradation of the hydrogel matrix prior to the replacement by autologous tissue can affect mechanical stability and therapeutic outcomes. Therefore, while a low friction coefficient is generally ensured, achieving higher compressive stiffness and adequate stability for cartilage repair remains a challenge. A promising approach to overcome these limitations is the combination of advanced hydrogels (including nanomaterials and multi-materials interpenetrating hydrogel networks) to develop composites with superior properties, including self-healing capabilities.

Self-healing hydrogels have the unique ability to autonomously repair themselves upon damage, making them valuable as tissue adhesives and wound-healing patches. Albumin-based hydrogels are an example of self-healing hydrogels, offering versatility, ease of gelation, pH and temperature responsiveness. While albumin has primarily been researched for its use as a drug delivery carrier, Meng et al. discuss its potential applications in the field of soft tissue engineering, along with the functional behavior and preparation methods of different hydrogel structures. The mechanical properties of albumin-based hydrogels are tunable, with tensile strength up to hundreds kPa and compressive strength ranging from about 0.1 to 10 MPa. Their self-healing properties can be quantitatively evaluated by comparing the tensile stress-strain behavior of original and healed samples, revealing an increase of tensile strength with the healing time. These smart features make them suitable for the development of both injectable hydrogels, if softer, and scaffolds, if stiffer.

Protein-based hydrogels can also be used as 3D culture systems, in order to mimic a complex extracellular environment with a highly porous topography at the nanoscale. In this regard, Kozlowski et al. demonstrate the efficacy of two novel protein-based hydrogels as 3D medium for supporting the survival and growth of primary endocrine cells and endocrine progenitor cells. In this context, the control of the mechanical properties of the 3D culture system is fundamental. The chemical and mechanical properties of protein-based hydrogels can be tuned to better control the microenvironment surrounding the cells, providing new insights on mechanotransduction phenomena in cell differentiation and maturation.

Another biopolymer used to develop hydrogels with customizable mechanical properties is gelatin, being one of the main ECM components in soft tissues. In order to optimize the preparation method of different formulations, Yousefi-Mashouf et al. compare the mechanical behavior of neat gelatin hydrogels and gelatin covalently cross-linked with glutaraldehyde at different concentrations, in several loading conditions, i.e., tension, compression, shear, under finite strains, cyclic loading and different strain rates. This extended test protocol allows to identify the effect of the cross-linker concentration on the mechanical strength and stiffness of gelatin, and can be considered as a basis for the selection of specific cross-linking conditions to obtain desired mechanical properties in view of target tissue repair applications.

In order to better understand the mechanical and structural properties of hydrogels for soft tissue replacement, experimental mechanical testing can be associated to constitutive and computational modeling. Kainz et al. adopt this approach to characterize the poro-viscoelastic behavior of a polyvinyl alcohol-based hydrogel, tailored to mimic the compressive behavior of brain tissue. In general, hydrogels are modeled as composed of an elastic solid matrix and a fluid phase: the time-dependent mechanical behavior is controlled by the flow of the fluid phase through the polymer network under compressive loading. Moreover, viscoelastic phenomena occur in physically cross-linked hydrogels. The distinction between the viscoelastic relaxation in the polymer network and the fluid time-dependent response is challenging from an experimental point of view, as it is necessary to clarify the role of absorbed and bounded water in the hydrogel matrix and the effect of compressive loads on the amount and flow of the liquid phase.

While addressing several different themes related to the mechanical behavior of hydrogels for soft tissue replacement and regeneration, this Research Topics aims to highlight the importance of investigating and understanding mechanical aspects, both with experimental and computational methods, which could improve the functionality of hydrogels *in vivo*, in the short and long-term performances.

